# Anxiety, depression, and sleep quality among breast cancer patients in North China: Mediating roles of hope and medical social support

**DOI:** 10.1007/s00520-023-07972-4

**Published:** 2023-08-08

**Authors:** Wenjuan Zhu, Jinnan Gao, Jun Guo, Linying Wang, Wanling Li

**Affiliations:** 1 Breast Surgery Department, Shanxi Bethune Hospital, Shanxi Academy of Medical Sciences, Tongji Shanxi Hospital, Third Hospital of Shanxi Medical University, Shanxi Province, Taiyuan, 030032 China; 2 Comprehensive Medical Department, Tongji Hospital, Tongji Medical College, Huazhong University of Science and Technology, Hubei Province, Wuhan, 430030 China; 3 Nursing Department, Shanxi Bethune Hospital, Shanxi Academy of Medical Sciences, Tongji Shanxi Hospital, Third Hospital of Shanxi Medical University, Shanxi Province, Taiyuan, 030032 China; 4 Tongji Hospital, Tongji Medical College, Huazhong University of Science and Technology, Hubei Wuhan, 430030 People’s Republic of China

**Keywords:** Anxiety, Depression, Sleep quality, Breast cancer, Mediating role, Social support

## Abstract

**Background:**

Sleep disorders are highly prevalent among breast cancer patients and have a detrimental impact on their quality of life. This study aims to investigate the serial multiple mediating roles of social support and hope in the relationship between anxiety, depression, and sleep quality in breast cancer patients.

**Methods:**

A cross-sectional study was conducted in China from October 2021 to February 2022. A total of 315 breast cancer patients were assessed using self-reported questionnaires, including the Pittsburgh Sleep Quality Index (PSQI), Medical Outcomes Study Social Support Survey (MOS-SSS), Herth Hope Index (HHI), and Hospital Anxiety and Depression Scale (HADS). Mediation analysis was performed using the R Statistical Software.

**Results:**

Sleep quality exhibited a negative correlation with hope and medical social support (*P* < 0.01), and a positive correlation with anxiety and depression (*P* < 0.01). Anxiety and depression accounted for 18.8% and 12.8% of the variance in sleep quality, respectively. Bootstrap analyses of the anxiety-medical social support-hope-sleep quality pathway indicated the presence of direct effects [B = 0.331, 95%CI (0.215, 0.493)] and indirect effects of anxiety on sleep quality mediated solely by medical social support [B = 0.054, 95%CI (0.015, 0.108)] and hope [B = 0.041, 95%CI (0.018, 0.073)], as well as combined effects [B = 0.012, 95%CI (0.004, 0.025)]. Similarly, the depression-medical social support-hope-sleep quality pathway revealed direct effects [B = 0.235, 95%CI (0.104, 0.372)] and indirect effects of depression on sleep quality mediated solely by medical social support [B = 0.078, 95%CI (0.016, 0.150)] and hope [B = 0.049, 95%CI (0.018, 0.086)], as well as combined effects [B = 0.017, 95%CI (0.004, 0.034)].

**Conclusions:**

This research validates the hypothesis that medical social support and hope serve as mediators between anxiety, depression, and sleep quality in breast cancer patients. Interventions targeting anxiety, depression, medical social support, and hope have the potential to effectively enhance sleep quality.

**Supplementary Information:**

The online version contains supplementary material available at 10.1007/s00520-023-07972-4.

## Introduction

Breast cancer, accounting for 11.7% of all new cancer cases in 2020, has emerged as the most prevalent cancer type [1]. Among the various side effects of breast cancer treatment, sleep disturbances significantly impact patients' daily lives, resulting in diminished quality of life, disruptions in regular activities, memory decline, and impaired interpersonal relationships [2]. The prevalence of sleep disorders in breast cancer patients ranges from 39.5% to 69.0%, depending on assessment timing and measurement tools [3]. Compared to malignancies of the prostate, gynecology, head and neck, urinary tract, and gastrointestinal tract, breast cancer exhibits the highest incidence of sleep disturbances [4]. Sleep disorders exert a profound influence on patients' behavioral, psychological, and emotional functions [5]. Certain studies have revealed that reduced sleep duration is associated with an increased risk of breast cancer recurrence, breast cancer-specific mortality, and overall mortality [6]. However, sleep disorders in breast cancer patients remain inadequately managed due to reduced hospital stays resulting from DRG (Diagnosis Related Groups) implementation, with healthcare providers prioritizing the management of treatment-related adverse effects. Therefore, effective screening and improvement of sleep quality are crucial for enhancing health outcomes in breast cancer patients undergoing chemotherapy (BCUC). Furthermore, it is imperative to investigate the factors influencing sleep disorders in BCUC.In conclusion, sleep disorders are prevalent in breast cancer patients and have an impact on their health, thus requiring the attention of healthcare professionals.

Several studies have indicated that sleep quality in BCUC is influenced by various factors, including sociodemographic factors such as age, BMI, and household income, as well as treatment-related factors such as surgery and chemotherapy [7]. Negative emotions, such as anxiety and depression, have also been shown to impact sleep quality in previous research [8]. Previous studies have demonstrated a relationship between sleep problems and anxiety-depression [9, 10]. Anxiety and depression can lead to hyperfunction of the hypothalamic–pituitary–adrenal axis, increased sympathetic excitability, and elevated circulating levels of norepinephrine and cortisol, resulting in a heightened state of arousal for patients [11], which in turn affects their sleep. A meta-analysis has demonstrated that greater social support can improve sleep quality in patients [12], and this finding has been supported by another study [13]. Several studies have also shown a positive correlation between sleep quality and hope [14, 15]. It is clear from the above that sleep disturbances in BCUC are related to many factors such as demographic characteristics, disease factors, anxiety and depression, hope and social support, among others.

Global prevalence rates of anxiety and depression in breast cancer patients have been reported as 41.9% [16] and 32.0% [17], respectively, according to systematic evaluation studies with large samples. Anxiety and depression frequently co-occur in patients [10], manifesting as reduced interest, poor concentration, loss of appetite, restlessness, despair, and even suicidal ideation [18]. All these psychological disorders can negatively impact disease management and reduce health outcomes. Therefore, it is crucial to conduct clinical evaluations of anxiety and depression in patients. Research has established an adverse association between anxiety, depression, and hope [17, 19]. Studies on advanced cancer patients have shown that social support can mediate the relationship between hope and depression [20]. A survey of young breast cancer patients has demonstrated a negative correlation between anxiety, depression, and social support [21]. To sum up, anxiety and depression often co-exist in breast cancer patients and affect the quality of life, related to sleep quality, hope level, and social support.

Social support [22] refers to the material and emotional assistance received from various social connections within a social network. Social support from family, friends, and significant others can enhance personal health and coping strategies, thereby mitigating the impact of illness-induced stress on individuals, as demonstrated by Zhao et al. [23]. Kugbey et al. survey of 205 breast cancer patients revealed that social support mediates the effect of anxiety and depression on the quality of life of breast cancer patients [24]. Both social support and hope are protective factors and may interact according to a protective-protective model [25]. Therefore, social support and hope may affect anxiety and depression through their combined effect. Social support can establish and expand resources to enhance the role of hope [26]. Referring to the above, social support as a protective factor can alleviate anxiety and depression, enhance hope, and promote patients' health.

Hope is an internal strength that assists patients in coping with suffering and achieving their goals, while also impacting their recovery, prognosis, and physical and mental health [27]. It is worth noting that Snyder et al. [26] have indicated that hope comprises two components: (1) Agency, which reflects assessments of one's capacity to initiate and sustain goal-directed motivation; and (2) Pathways, which involves contemplating techniques or approaches for achieving desired objectives. Thus, hope is an essential factor for cancer patients. When patients encounter obstacles, hope drives them to have greater determination in attaining their goals and in planning how to do so. Consequently, patients are better able to adjust to these stressors, and those with high levels of hope spend less time ruminating on their disease-related suffering [28]. Furthermore, hope can modify psychological symptoms through the assessment and response to stress. In summary, hope is also a protective factor to help patients adapt to stress and adjust to psychological discomfort.

Understanding the pathogenesis of sleep disorders can provide guidance for formulating intervention plans for sleep disorders in the future. While previous research has demonstrated the interaction among the aforementioned factors, empirical testing of their impact on sleep disorders within a unified model has been lacking. Therefore, a serial multiple mediating model was employed to examine the relationships between anxiety, depression, social support, hope, and sleep quality. This study aims to test the following hypotheses: (1) Anxiety and depression are associated with sleep quality; (2) Medical social support mediates the relationship between anxiety, depression, and sleep quality; (3) Hope mediates the relationship between anxiety, depression, and sleep quality; and (4) Medical social support and hope jointly mediate the relationship between anxiety, depression, and sleep quality.

## Methods

### Study design

A cross-sectional study was conducted at the Breast Surgery Department of Shanxi Baiqiuen Hospital, a tertiary hospital in Shanxi, China, from October 2021 to February 2022.

### Setting and participants

The inclusion criteria for participants were as follows: (1) age ≥ 18 years; (2) female; (3) initial diagnosis of breast cancer and awareness of the disease; (4) ability to communicate verbally and read independently; (5) absence of neurological and psychiatric disorders. The exclusion criteria were: (1) prior diagnosis of a sleep disorder; (2) use of glucocorticoids or sedatives during the study period; (3) refusal to participate in the study. The study was approved by the Shanxi Baiqiuen Hospital Ethics Committee, and written informed consent was obtained from all patients.

### Data collection and questionnaires

Participants were selected using convenience sampling. Before administering the questionnaire, the study purpose was explained to the participants, and their informed consent was obtained. Two trained researchers conducted the study in the breast surgery ward. Participants received instructions on questionnaire completion and safety procedures, and they completed a paper-based Chinese questionnaire. The survey took approximately 20 to 30 min, and all questionnaires were collected on-site, checked for completeness, and any missing items were promptly addressed. In cases where medical records were unavailable, patients provided self-reported demographic and illness information. The collected information was double-checked and entered anonymously to ensure patient confidentiality. A third investigator reviewed 10% of the surveys for error correction.

#### Dependent variable: The Pittsburgh Sleep Quality Index (PSQI)

Consists of nineteen questions grouped into seven components: insomnia, subjective sleep quality, sleep duration, sleep disturbances, sleep efficiency, use of sleep medication, and daytime dysfunction. The total PSQI score ranges from 0 to 21, with a score of 7 indicating good sleep quality and a score of 8 or higher indicating poor sleep quality [29].

#### Mediator variable: Medical Outcomes Study Social Support Survey (MOS-SSS)

Measures the individual level of medical and social support and includes nineteen items and four subscales: emotional/informational support, tangible support, affectionate support, and positive social interaction. The items are scored on a 1–4 scale. The overall score ranges from 19 to 76, with higher scores indicating greater social support [30].

#### Mediator variable: The Herth Hope Index (HHI)

Consists of twelve statements with Likert scale responses: strongly disagree (1), disagree (2), agree (3), and strongly agree (4). The total score is the sum of the scores from the twelve items, ranging from 12 to 48. Scores from 12 to 23 indicate low hope, 24 to 35 indicate medium hope, and 36 to 48 indicate strong hope [31].

#### Independent variable: Hospital Anxiety and Depression Scale (HADS)

Consists of two subscales, anxiety and depression, with seven items each. The scores from the corresponding questions in each subscale are summed to obtain anxiety and depression scores. An anxiety score of 8 or below indicates the absence of anxiety symptoms, while a score of 8 or higher indicates the presence of anxiety symptoms. Similarly, a depression score of 8 or below indicates the absence of depressive symptoms, while a score of 8 or higher indicates the presence of depressive symptoms [32].

### Statistical analyses

Data analysis was performed using SPSS 22.0 and R Statistical Software (version 4.2.1; R Foundation for Statistical Computing, Vienna, Austria). Categorical data were described using frequencies and percentages, while continuous data were presented as means with standard deviations (SD). Bivariate analyses were conducted to examine the relationships between all variables. A multiple linear regression model was developed to determine the relationships between anxiety, depression, hope, medical social support, and sleep quality, while adjusting for patients' sociodemographic characteristics. A chain mediation model was employed to validate and confirm the path relationships. Demographic information influencing sleep quality was included as a covariate. In order to investigate the impact of anxiety and depression on sleep quality, different models were constructed. Sleep quality was assigned as the dependent variable, while anxiety and depression served as the independent variables. Additionally, medical social support and hope were included as mediating variables.To assess the significance of the mediating effect, a bias-corrected bootstrap method was used to establish 95% bias-corrected confidence intervals, with 5000 data replication samples. Goodness-of-fit was tested using structural equation modeling. A *p*-value of < 0.05 (two-sided) was considered statistically significant.

## Results

### Demographics and clinical characteristics (Table 1)

**Table 1:** The demographic and clinical characteristics of patients with breast cancer

Characteristic	N (%)
Age (year)	
~ 45	105(33.3)
45 ~ 55	108(34.3)
55 ~	102(32.4)
Marriage	
Married	298(94.2)
Separated/divorced	10(3.2)
Widowed	7(2.2)
Income (RMB/month)	
> 8,000	34(10.8)
5,000–8,000	49(15.6)
2000–5000	148(47.0)
0–2,000	84(26.7)
Education	
College	98(31.1)
High school	56(17.8)
Middle school or below	161(51.1)
Surgery	
No	78(75.2)
Yes	237(24.8)
T stage	
1	120(38.1)
2	156(49.5)
≥ 3	39(12.4)
N stage	
0	114(36.2)
1	118(37.5)
≥ 2	83(26.3)
M stage	
0	299(94.9)
1	16(5.1)
Anxiety	
No(A < 8)	233(74.0)
Yes(A ≥ 8)	82(26.0)
Depression	
No(D < 8)	247(78.4)
Yes(D ≥ 8)	68(21.6)
Sleep quality	
Good (PSQI < 8)	178(56.5)
Poor (PSQI ≥ 8)	137(46.5)
Hope level	
Low (12–23)	0(0)
Medium (24–35)	118(37.5)
High (36–48)	197(62.5)
Medical social support	M(SD)
Total support	67.53(16.18)
emotional/informational support	26.44(7.41)
Tangible support	15.52(3.65)
Affectionate support	11.46(3.78)
Positive social interaction support	14.12(3.78)

A total of 330 questionnaires were distributed in this study, out of which 315 were deemed valid, resulting in a valid return rate of 95.5%. Twelve patients did not meet the exclusion criteria, and three surveys were found to be faulty. The age range of the 315 breast cancer patients was 25–72 years, with a mean age of 49.76 ± 10.26 years.

### Participants’ scores on the Hospital Anxiety and Depression Scale, Pittsburgh Sleep Quality Index, Herth Hope Scale, and Medical Social Support Scale (Table 1)

According to the findings of this study, 82 respondents (26.0%) reported anxiety scores ≥ 8, and 68 (21.6%) had depression scores ≥ 8. Furthermore, 137 respondents (43.5%) experienced sleep disturbance. Among the participants, 197 (62.5%) breast cancer patients exhibited a high level of hope. As there was no specific scoring system for the MOS-SSS scale, and the scores followed a normal distribution, they were presented as mean ± standard deviation values, the mean score of the total medical social support score was 67.53(SD = 16.18, range = 0 to 95).

### Correlation between the study variables (Table 2)

**Table 2:** Spearman correlation analysis of the study variables

Variables	Sleep quality	Hope	Anxiety	Depression	Medical social support	Tangible support	Emotional/informational support	Positive social interaction support	Affectionate support
Sleep quality	1								
Hope	-0.288**	1							
Anxiety	0.429**	-0.246**	1						
Depression	0.349**	-0.259**	0.695**	1					
Medical social support	-0.256**	0.242**	-0.341**	-0.455**	1				
Tangible support	-0.210**	0.148**	-0.255**	-0.327**	0.814**	1			
Emotional/informational support	-0.243**	0.209**	-0.269**	-0.412**	0.939**	0.664**	1		
Positive social interaction support	-0.238**	0.252**	-0.379**	-0.451**	0.890**	0.661**	0.772**	1	
Affectionate support	-0.254**	0.255**	-0.379**	-0.453**	0.878**	0.691**	0.762**	0.797**	1

**P < 0.01

The results of Pearson correlation analysis revealed a significant positive correlation between sleep quality and anxiety (r = 0.429, *p* < 0.01), as well as between sleep quality and depression (r = 0.349, *p* < 0.01). Sleep quality demonstrated a significant negative correlation with hope (r = -0.288, *p* < 0.01) and with medical social support (r = -0.256, *p* < 0.01). Additionally, we examined the correlation between demographic characteristics and sleep quality, according to our analysis, only age exhibited a significant association with sleep quality and the results are provided in Supplementary Material (Stable 1). In subsequent mediation analysis, we further included age as a covariate in the model.

### Mediating effect of hope and medical social support (Table 3, Fig. 1)

**Table 3:** Direct and indirect effects and 95% confidence intervals for the models

Path	Effect	Boot SE	Boot LLCI	Boot ULCI	z	p
Anxiety → sleep quality						
Total effects	0.439	0.075	0.313	0.599	5.877	< 0.001
Direct effects	0.331	0.071	0.215	0.493	4.663	< 0.001
Indirect effects	0.108	0.029	0.058	0.172	3.662	< 0.001
Anxiety → Medical social support → Sleep quality	0.054	0.024	0.015	0.108	2.259	0.024
Anxiety → Hope → Sleep quality	0.041	0.014	0.018	0.073	2.895	0.004
Anxiety → Hope → Medical social support → Sleep quality	0.012	0.006	0.004	0.025	2.176	0.030
Depression → Sleep quality						
Total effects	0.379	0.060	0.266	0.500	6.333	< 0.001
Direct effects	0.235	0.069	0.104	0.372	3.421	< 0.001
Indirect effects	0.143	0.039	0.072	0.226	3.659	< 0.001
Depression → Medical social support → Sleep quality	0.078	0.034	0.016	0.150	2.269	0.023
Depression → Hope → Sleep quality	0.049	0.018	0.018	0.086	2.755	0.006
Depression → Hope → Medical social support → Sleep quality	0.017	0.008	0.004	0.034	2.146	0.032

Boot LLCI:Bootstrap 95% lower; Boot ULCI: Bootstrap 95% upper

**Fig. 1:**
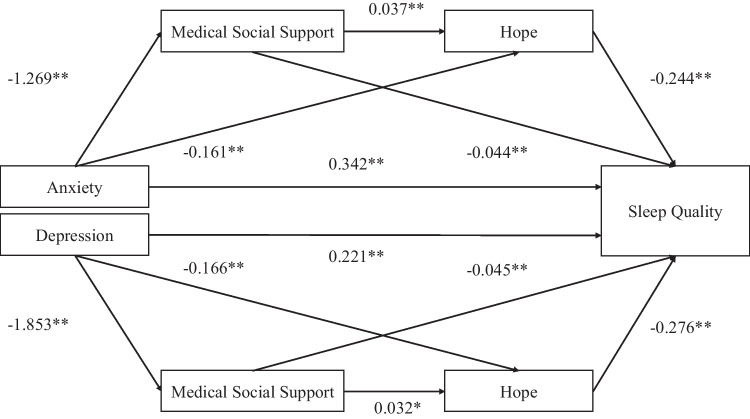
The observed path models. The beta values in parenthesis indicate the direct effects of the predictors on sleep quality. Path coefficients are standardized estimates. *p < 0.05; **p < 0.01 level; ***p < 0.001, n.s not significant at the .05 level

After controlling for age, which was significantly associated with the study variables in bivariate analyses,the fitness indicators for the anxiety-hope-medical social support-sleep quality model were as follows: RMSEA = 0.095, TLI = 0.786, CFI = 0.914. For the depression-hope-medical social support-sleep quality model, the indicators were: RMSEA = 0.066, TLI = 0.904, CFI = 0.961. The coefficients for all paths are shown in Fig. 1.

The results show that the 95% confidence intervals (CIs) for the total, direct, and indirect effects of the model did not include 0, indicating that the mediating effect is significant.Path analysis revealed that the direct effect of anxiety on sleep quality was 0.331, while the total indirect effect value was 0.108, and the total effect value was 0.439. Similarly, the direct effect of depression on sleep quality was 0.235, and the total indirect effect value was 0.143, and the total effect value was 0.379.

## Discussion

This study aimed to investigate the relationship between anxiety, depression, hope, medical social support, and sleep quality in breast cancer patients. The findings indicate that these variables are interconnected, with anxiety, depression, hope, and medical-social support all being associated with sleep quality. Two chain mediation models were constructed to examine the impact of anxiety and depression on sleep quality. The models demonstrated a good fit to the data and confirmed all assumptions. The direct effects of anxiety and depression on sleep quality accounted for 18.8% and 12.8% of the total effects, respectively, and both effects were statistically significant. Furthermore, medical social support and hope were found to partially mediate the relationship between anxiety, depression, and sleep quality, explaining less than half of the total effect. Exploring factors that influence sleep quality in breast cancer patients is critical for improving their overall well-being.

### The association between anxiety, depression and sleep quality

The finding that 46.6% of breast cancer patients in this study had a Pittsburgh Sleep Quality Index (PSQI) score ≥ 8 suggests that this population experiences sleep problems, highlighting the need for sleep management in breast cancer chemotherapy patients. Sleep disturbances can lead to various medical and mental illnesses, resulting in cognitive decline, decreased appetite, and weakened immunity [24]. Sleep problems not only impact patients' quality of life but also affect the metabolism of substances like glycogen storage [33]. This study found a positive correlation between anxiety, depression, and sleep quality in breast cancer patients. Specifically, lower levels of anxiety and depression were associated with a reduced likelihood of sleep disturbance, consistent with the findings of Carreira [10].Miloseva L [34] also reported a negative correlation between anxiety, depression, and sleep quality in 152 preoperative breast cancer patients. Since patients often experience anxiety and depression concurrently [35], it is crucial to detect early signs of psychological distress in clinical treatment and provide guidance and support, such as Cognitive-Behavioral Therapy, to improve sleep quality and enhance patients' resilience.

### Mediating role of medical social support on anxiety, depression, and sleep quality

The study clearly indicates that medical social support plays a significant mediating role between anxiety, depression, and sleep quality. Social support acts as a stress buffer between negative events (e.g., illness) and anxiety-depression, making it a significant predictor [36]. The buffering model suggests that social support protects individuals from the potentially harmful effects of stressful events [35], a concept supported by additional research [37]. The positive impact of social support is influenced by personal perception. Perceived social support [34] refers to the subjective assessment of how individuals perceive the availability of economic, emotional, and general assistance from relatives and close friends. Individual perceptions determine the positive influence of social support. Perceived social support has been found to be negatively correlated with psychological discomfort and suicidal thoughts [13], and the ability to perceive social support is diminished in patients with anxiety and depression [38]. A previous study [39] demonstrated that social support can provide diverse information sources and increase the availability of information that encourages health-related behaviors, including maintaining healthy sleep patterns. Medical social support can provide a secure sleeping environment, reducing vigilance and alertness, thereby facilitating adequate rest.

### Mediating role of hope on anxiety, depression, and sleep quality

The mediation model reveals that hope has a significant indirect effect on sleep quality, both independently and through the mediating role of medical social support. With the emergence of positive psychology, researchers have focused more on the impact of positive psychological factors on health. Hope [40] is a positive psychological construct and a powerful force that motivates patients to transcend the realities of their life circumstances and find meaning in their lives. According to Kaleta et al. [28], hope can assist cancer patients in coping with their condition and promote positive behaviors that alleviate depression. Studies [40] indicate that breast cancer patients with high levels of hope possess greater disease knowledge and engage in more informed medical decision-making than those with low levels of hope. Patients with high levels of hope also exhibit positive psychological changes following a cancer diagnosis, such as finding new purpose, deepening their spirituality, and engaging in positive activities. Hope [41] not only leads to a significantly stronger immune system but also improves overall quality of life and health. Hope can be a vital component in the treatment process for cancer patients, as it significantly alleviates pain, helps overcome obstacles, improves mood, and enhances sleep quality.

### Chain-mediated role of medical social support and hope on anxiety, depression, and sleep quality

Path analysis revealed that the effects of anxiety and depression on sleep quality remained significant even after considering the influence of medical social support and hope, indicating that anxiety and depression indirectly affect sleep quality through the mediating effects of medical social support and hope. The levels of medical social support and hope mediate the relationship between anxiety, depression, and sleep quality. Within the framework of resilience theory, Fergus and Zimmerman [40] proposed a protective-protective model to describe the mechanisms of influence between two protective variables. They suggest that these protective variables interact with each other to produce results. Building on this theoretical framework, hope and social support may interact with each other concerning sleep quality in breast cancer patients. For example, Snyder's study [26] demonstrated that hope levels can be enhanced by building and strengthening social support resources. Consistent with Chen's findings [25], the present study reveals that the depression-medical social support-hope-sleep quality pathway demonstrates how social support and hope collectively influence sleep quality as coping factors. Sufficient social support can promote positive psychological resources, such as hope. Hope represents an internal positive component, while social support serves as an external positive factor. Medical social support, as an external positive factor, is more amenable to change, providing a valuable clue for clinical care. In our clinical practice, assessing patients' social support and providing appropriate assistance based on the results can lead to improved health outcomes. However, the moderating role of medical social support and hope is not fully understood, and further research is needed to explore the association between anxiety, depression, sleep treatment, and other influential factors in breast cancer patients.

### Limitations

This research has several limitations. Firstly, the cross-sectional design used in this study does not establish a direct causal relationship between the four factors. All four variables are dynamic and subject to change over time. However, the cross-sectional approach only captures the current situation, which limits the reliability of the study's model. Furthermore, convenience sampling was employed, which lacks sample representativeness and restricts the generalizability of the model. Future research should consider longitudinal studies and random sampling to address these limitations. Secondly, the sole source of information was the participants themselves, which may introduce information bias. Other cognitive factors and biological parameters, such as consciousness, pain perception, and functional capacity, may potentially influence the mediating effects of the model. However, these factors were not considered in this study. Incorporating these variables into future research would be beneficial.

## Conclusion

The present study showed a high prevalence of sleep disturbance in BCUC and demonstrated that anxiety and depression could directly and indirectly affect sleep quality through medical social support and hope in BCUC. Our findings suggest that hope and social support as positive factors have great potential to improve the quality of sleep of patients. By mobilizing various sources of social support, such as family, friends, community, and hospitals, we can effectively address the sleep issues faced by patients. Future research can explore the variable effects of social support from different sources on patients' sleep quality.

## Supplementary Information

Below is the link to the electronic supplementary material.

Supplementary file1 (DOCX 15 KB)

Supplementary file2 (DOCX 15 KB)

## Data Availability

All relevant data are within the manuscript and its additional files. The data are available from the corresponding author on reasonable request.
