# Supplementary material for: Anxiety, depression, and sleep quality among breast cancer patients in North China: Mediating roles of hope and medical social support
Source: Support Care Cancer. 2023 Aug 8;31(9):514. doi: 10.1007/s00520-023-07972-4 (PMC10409667; doi:10.1007/s00520-023-07972-4)
Supplement: Supplementary file 1 — Supplementary file1 (DOCX 15 KB) [file 520_2023_7972_MOESM1_ESM.docx]

**Supplementary Table 1 Association between Demographic and Clinical Information with Sleep Quality**

| **Characteristic** | **n** | **Sleep Quality Score** | **Sleep Quality (%)** | |  | ***P*** | |
| --- | --- | --- | --- | --- | --- | --- | --- |
|  |  |  | **Good** | **Poor** |  | **Kruskal-Wallis** | **χ^2^** |
| Age (year) |  |  |  |  |  |  |  |
| ~45 |  | 5.0 [4.0, 8.0] | 35 (33.3%) | 70 (66.7%) |  | 0.003 | 0.017 |
| 45~55 | 108(34.3%) | 7.0 [4.0, 10.0] | 48 (44.4%) | 60 (55.6%) |  |  |  |
| 55~ | 102(32.4） | 8.0 [5.0, 11.0] | 54 (52.9%) | 48 (47.1%) |  |  |  |
| Marriage |  |  |  |  |  | 0.822 | 0.484 |
| Married | 298(94.2%) | 7.0 [4.0, 10.0] | 132 (44.3%) | 166 (55.7%) |  |  |  |
| Separated/divorced | 10(3.2%) | 6.5 [5.2, 8.5] | 3 (30.0%) | 7 (70.0%) |  |  |  |
| Widowed | 7(2.2%) | 6.0 [5.5, 7.0] | 2 (28.6%) | 5 (71.4%) |  |  |  |
| Income (RMB/month) |  |  |  |  |  | 0.083 | 0.078 |
| ＞8,000 | 34(10.8%) | 5.0 [4.0, 6.8] | 8 (23.5%) | 26 (76.5%) |  |  |  |
| 5,000-8,000 | 49(15.6%) | 6.0 [5.0, 11.0] | 20 (40.8%) | 29 (59.2%) |  |  |  |
| 2000-5000 | 148(47.0%) | 7.0 [4.0, 10.0] | 69 (46.6%) | 79 (53.4%) |  |  |  |
| 0-2,000 | 84(26.7%) | 7.0 [5.0, 10.0] | 40 (47.6%) | 44 (52.4%) |  |  |  |
| Education |  |  |  |  |  | 0.810 | 0.657 |
| College | 98(31.1%) | 6.0 [5.0, 9.8] | 39 (39.8%) | 59 (60.2%) |  |  |  |
| High school | 56(17.8%) | 7.0 [4.0, 10.0] | 26 (46.4%) | 30 (53.6%) |  |  |  |
| Middle school or below | 161(51.1%) | 7.0 [4.0, 10.0] | 72 (44.7%) | 89 (55.3%) |  |  |  |
| Surgery |  |  |  |  |  | 0.810 | 0.879 |
| No | 78(75.2%) | 7.0 [4.0, 9.8] | 35 (44.9%) | 43 (55.1%) |  |  |  |
| Yes | 237(24.8%) | 6.0 [4.0, 10.0] | 102 (43.0%) | 135 (57.0%) |  |  |  |
| T stage |  |  |  |  |  | 0.377 | 0.544 |
| 1 | 120(38.1%) | 7.0 [5.0, 10.0] | 56 (46.7%) | 64 (53.3%) |  |  |  |
| 2 | 156(49.5%) | 6.0 [4.0, 9.0] | 63 (40.4%) | 93 (59.6%) |  |  |  |
| ≥3 | 39(12.4%) | 7.0 [4.5, 9.5] | 18 (46.2%) | 21 (53.8%) |  |  |  |
| N stage |  |  |  |  |  | 0.108 | 0.312 |
| 0 | 114(36.2%) | 7.0 [5.0, 11.0] | 56 (49.1%) | 58 (50.9%) |  |  |  |
| 1 | 118(37.5%) | 6.0 [4.0, 9.0] | 47 (39.8%) | 71 (60.2%) |  |  |  |
| ≥2 | 83(26.3%) | 6.0 [4.0, 9.0] | 34 (41.0%) | 49 (59.0%) |  |  |  |
| M stage |  |  |  |  |  | 0.668 | 0.812 |
| 0 | 299(94.9%) | 7.0 [4.0, 10.0] | 131 (43.8%) | 168 (56.2%) |  |  |  |
| 1 | 16(5.1%) | 7.0 [3.8, 9.2] | 6 (37.5%) | 10 (62.5%) |  |  |  |

To examine the relationship between demographic characteristics, clinical information, and sleep quality in the general population, we initially conducted a rank-sum test to analyze the association between the raw scores of sleep quality and the aforementioned variables, based on the results of a normality test. Subsequently, we categorized sleep quality into "good" and "poor" groups using a cutoff of 8, and employed a chi-square test to investigate the relationship between sleep quality and the aforementioned variables. According to our analysis, only age group exhibited a significant association with sleep quality. In subsequent mediation analysis, we further included age as a covariate in the model.
