# Supplementary material for: Anxiety, depression, and sleep quality among breast cancer patients in North China: Mediating roles of hope and medical social support
Source: Support Care Cancer. 2023 Aug 8;31(9):514. doi: 10.1007/s00520-023-07972-4 (PMC10409667; doi:10.1007/s00520-023-07972-4)
Supplement: Supplementary file 2 — Supplementary file2 (DOCX 15 KB) [file 520_2023_7972_MOESM2_ESM.docx]

**Supplementary Table 1 Association between Demographic and Clinical Information with Sleep Quality**

| Item | Sleep Quality | Medical Social Support | Hope | Sleep Quality |
| --- | --- | --- | --- | --- |
| **Model 1** |  |  |  |  |
| Intercept | 7.400***(0.366) | 68.663 ***(1.494) | 34.545 ***(0.830) | 7.497 *** (0.351) |
| Age: ~45 | -1.098 *(0.521) | -1.536(2.127) | 0.321 (0.424) | -1.101 * (0.500) |
| Age:55~ | 1.174 * (0.525) | -1.906(2.146) | -0.301 (0.428) | 0.999 * (0.505) |
| Anxiety | 0.449 *** (0.057) | -1.269 *** (0.234) | -0.161 **(0.049) | 0.342 *** (0.058) |
| Medical Social Support |  |  | 0.037 ** (0.011) | -0.044 ** (0.014) |
| Hope |  |  |  | -0.244 *** (0.067) |
| R^2^ | 0.196 | 0.088 | 0.093 | 0.265 |
| Adj. R^2^ | 0.188 | 0.080 | 0.081 | 0.253 |
| **Model 2** |  |  |  |  |
| Intercept | 7.478 *** (0.379) | 68.583 *** (1.423) | 34.835 *** (0.867) | 7.530 *** (0.363) |
| Age: ~45 | -0.915(0.540) | -2.337 (2.028) | 0.236(0.425) | -0.977 (0.519) |
| Age:55~ | 0.868(0.544) | -0.837 (2.042) | -0.188 (0.427) | 0.770 (0.521) |
| Depression | 0.368 *** (0.062) | -1.853 *** (0.231) | -0.166 ** (0.053) | 0.221 ***(0.066) |
| Medical Social Support |  |  | 0.032 ** (0.012) | -0.045 **(0.015) |
| Hope |  |  |  | -0.276 *** (0.069) |
| R^2^ | 0.136 | 0.173 | 0.090 | 0.213 |
| Adj. R^2^ | 0.128 | 0.165 | 0.078 | 0.200 |

* p < 0.05, ** p < 0.01, *** p < 0.001.
